# Effects of dapagliflozin in heart failure with reduced ejection fraction and chronic obstructive pulmonary disease: an analysis of DAPA‐HF

**DOI:** 10.1002/ejhf.2083

**Published:** 2021-01-18

**Authors:** Pooja Dewan, Kieran F. Docherty, Olof Bengtsson, Rudolf A. de Boer, Akshay S. Desai, Jaroslaw Drozdz, Nathaniel M. Hawkins, Silvio E. Inzucchi, Masafumi Kitakaze, Lars Køber, Mikail N. Kosiborod, Anna Maria Langkilde, Daniel Lindholm, Felipe A. Martinez, Béla Merkely, Mark C. Petrie, Piotr Ponikowski, Marc S. Sabatine, Morten Schou, Mikaela Sjöstrand, Scott D. Solomon, Subodh Verma, Pardeep S. Jhund, John J.V. McMurray

**Affiliations:** ^1^ BHF Cardiovascular Research Centre University of Glasgow Glasgow UK; ^2^ Late Stage Development, Cardiovascular, Renal, and Metabolism, BioPharmaceuticals R&D AstraZeneca Gothenburg Sweden; ^3^ Department of Cardiology University Medical Centre and University of Groningen Groningen The Netherlands; ^4^ Division of Cardiovascular Medicine Brigham and Women's Hospital Boston MA USA; ^5^ Department of Cardiology Medical University of Lodz Lodz Poland; ^6^ Division of Cardiology University of British Columbia Vancouver Canada; ^7^ Section of Endocrinology Yale University School of Medicine New Haven CT USA; ^8^ Cardiovascular Division of Medicine National Cerebral and Cardiovascular Centre Osaka Japan; ^9^ Rigshospitalet Copenhagen University Hospital Copenhagen Denmark; ^10^ Saint Luke's Mid America Heart Institute University of Missouri Kansas City MO USA; ^11^ National University of Cordoba Cordoba Argentina; ^12^ Heart and Vascular Centre Semmelweis University Budapest Hungary; ^13^ Wroclaw Medical University Wroclaw Poland; ^14^ TIMI Study Group Brigham and Women's Hospital and Harvard Medical School Boston MA USA; ^15^ Department of Cardiology Gentofte University Hospital Copenhagen Denmark; ^16^ St Michael's Hospital University of Toronto Toronto Canada

**Keywords:** Heart failure, Dapagliflozin, Chronic obstructive pulmonary disease

## Abstract

**Aims:**

Chronic obstructive pulmonary disease (COPD) is an important comorbidity in heart failure (HF) with reduced ejection fraction (HFrEF), associated with worse outcomes and often suboptimal treatment because of under‐prescription of beta‐blockers. Consequently, additional effective therapies are especially relevant in patients with COPD. The aim of this study was to examine outcomes related to COPD in a *post hoc* analysis of the Dapagliflozin And Prevention of Adverse‐outcomes in Heart Failure (DAPA‐HF) trial.

**Methods and results:**

We examined whether the effects of dapagliflozin in DAPA‐HF were modified by COPD status. The primary outcome was the composite of an episode of worsening HF or cardiovascular death. Overall, 585 (12.3%) of the 4744 patients randomized had a history of COPD. Patients with COPD were more likely to be older men with a history of smoking, worse renal function, and higher baseline N‐terminal pro B‐type natriuretic peptide, and less likely to be treated with a beta‐blocker or mineralocorticoid receptor antagonist. The incidence of the primary outcome was higher in patients with COPD than in those without [18.9 (95% confidence interval 16.0–22.2) vs. 13.0 (12.1–14.0) per 100 person‐years; hazard ratio (HR) for COPD vs. no COPD 1.44 (1.21–1.72); *P* < 0.001]. The effect of dapagliflozin, compared with placebo, on the primary outcome, was consistent in patients with [HR 0.67 (95% confidence interval 0.48–0.93)] and without COPD [0.76 (0.65–0.87); interaction *P*‐value 0.47].

**Conclusions:**

In DAPA‐HF, one in eight patients with HFrEF had concomitant COPD. Participants with COPD had a higher risk of the primary outcome. The benefit of dapagliflozin on all pre‐specified outcomes was consistent in patients with and without COPD.

Clinical Trial Registration: ClinicalTrials.gov ID NCT03036124.

## Graphical abstract

Chronic obstructive pulmonary disease (COPD) in the DAPA‐HF trial: patient characteristics and clinical outcomes. AE, adverse event; CI, confidence interval; KCCQ‐TSS, Kansas City Cardiomyopathy Questionnaire total symptom score; MRA, mineralocorticoid receptor antagonist.

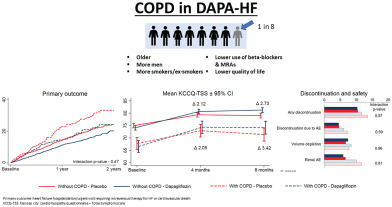

## Introduction

Chronic obstructive pulmonary disease (COPD) has a higher prevalence in patients with heart failure (HF) than in the general population.^1^, ^2^, ^3^ While this most likely reflects the common aetiological role of smoking in each condition, inflammation and oxidative stress have also been postulated to play a role.^1^, ^2^, ^3^ COPD is important in HF for two principal reasons. First, HF patients with COPD have worse outcomes, including higher mortality, than HF patients without COPD.^1^, ^2^, ^3^, ^4^, ^5^, ^6^ Second, concomitant COPD has implications for the use of specific HF therapies.^1^, ^2^, ^3^, ^7^ Physicians may be reluctant to use beta‐blockers in HF patients with COPD, because of their bronchospastic effects, even though these drugs are lifesaving in patients with HF with reduced ejection fraction (HFrEF). Although still indicated and generally well tolerated in most patients with COPD, even in high‐risk settings, new evidence also shows that beta‐blockers can increase the risk of respiratory hospitalization in patients with more severe COPD.^8^, ^9^ It is also advised that hydralazine and isosorbide dinitrate are avoided in patients with COPD as this combination may lead to worsening gas exchange and hypoxaemia.^7^ Similarly, hypokalaemia induced by beta‐agonists, glucocorticoids and xanthine derivatives may make digoxin use more hazardous in patients with COPD, compared to those without and increase risk of arrhythmias.^7^, ^10^, ^11^

Consequently, there is a need for new therapies, both in addition to conventional treatment and, occasionally, as an alternative due to non‐tolerance of standard pharmacotherapies, in these high‐risk individuals with both HFrEF and COPD. Recently, specific sodium–glucose co‐transporter 2 inhibitors have demonstrated reduction in mortality and risk of HF hospitalization in patients with HFrEF.^12^, ^13^, ^14^ In this *post hoc* analysis, we examined the effects of dapagliflozin in HFrEF patients with and without COPD enrolled in the Dapagliflozin And Prevention of Adverse‐outcomes in Heart Failure (DAPA‐HF) trial.^12^, ^15^, ^16^

## Methods

DAPA‐HF was a randomized, double‐blind, placebo‐controlled, event‐driven trial in HFrEF patients.^12^, ^15^, ^16^ Efficacy and safety of dapagliflozin 10 mg once daily, plus standard care, was compared with matching placebo. The design, baseline characteristics, and primary results are published.^12^, ^15^, ^16^ The Ethics Committee of the 410 participating institutions (20 countries) approved the protocol, and all patients gave written informed consent. The investigation conforms with the principles outlined in the Declaration of Helsinki.^17^

### Study patients

Enrolment criteria included HF with left ventricular ejection fraction (LVEF) ≤40%, age ≥18 years, New York Heart Association (NYHA) functional class II–IV, elevated N‐terminal pro B‐type natriuretic peptide (NT‐proBNP), and optimal pharmacological and device therapy. The protocol required guideline‐recommended medications, including beta‐blocker, unless contraindicated/not tolerated. Key exclusion criteria included: symptomatic hypotension/systolic blood pressure <95 mmHg, estimated glomerular filtration rate (eGFR) <30 mL/min/1.73 m^2^(or rapidly declining renal function), and type 1 diabetes mellitus. No exclusions were related to COPD or asthma.

### Identification of chronic obstructive pulmonary disease and study follow‐up

An investigator‐reported history of COPD was identified from a check box on the case report form. No specific instructions were given in relation to diagnosis of COPD. Investigators reported diagnosis of asthma similarly. There were no respiratory disease or respiratory treatment‐related exclusions, although investigators were asked to exclude patients with another condition likely to lead to life expectancy of <2 years. After randomization, follow‐up visits occurred at 14, 60, 120, 240, 360 days and every 4 months thereafter.

### Study outcomes

The primary trial outcome was the composite of worsening HF (HF hospitalization or urgent visit for HF requiring intravenous therapy) or cardiovascular (CV) death, whichever occurred first. Pre‐specified secondary endpoints included HF hospitalization or CV death; HF hospitalizations (first and recurrent) and CV deaths; change from baseline to 8 months in Kansas City Cardiomyopathy Questionnaire total symptom score (KCCQ‐TSS)^18^; incidence of worsening renal function and all‐cause death (because of few renal events overall, this endpoint was not examined in the present analysis). In addition to the pre‐specified outcomes, we also analysed: (i) KCCQ overall summary score (KCCQ‐OSS) and KCCQ clinical summary score (KCCQ‐CSS), and (ii) non‐CV deaths, in view of the potential impact of COPD on quality of life and deaths from respiratory causes and infection. Pre‐specified safety analyses included any serious adverse event (AE), AEs related study drug discontinuation, AEs of interest and laboratory findings of note.

### Statistical analysis

The primary analysis examined patients with an investigator‐reported history of COPD, including a small number with concurrent asthma; patients with asthma alone were examined in supplementary analyses (online supplementary material).

Baseline characteristics were summarized as means (standard deviations), median (interquartile ranges), or percentages. Time‐to‐event data were evaluated using Kaplan–Meier estimates and Cox proportional‐hazards models, stratified by diabetes status, and adjusted for history of HF hospitalization (except for non‐CV and all‐cause death) and treatment‐group assignment. We used a semiparametric proportional‐rates model to analyse total (including recurrent) events, as previously described. We also adjusted the effect of COPD status in two additional models: Model 1 included age, sex, region, systolic blood pressure, history of atrial fibrillation, NYHA class III/IV, LVEF, log NT‐proBNP, eGFR and smoking status. Model 2 had additional adjustment for baseline beta‐blocker and mineralocorticoid receptor antagonist (MRA) prescription. We analysed mean change in KCCQ‐TSS from baseline to 8 months. Safety analyses were performed in randomized patients who had received at least one dose of dapagliflozin or placebo (8 of 4744 patients excluded). Interaction between COPD status and treatment effect on the occurrence of the pre‐specified safety outcomes was tested in a logistic regression model with an interaction term between baseline COPD status and treatment.

All analyses were conducted using Stata version 16.1 (Stata Corp., College Station, TX, USA). A *P*‐value <0.05 was considered statistically significant. 

## Results

Overall, 585 (12.3%) of 4744 patients randomized had COPD, 299 (12.6%) in the dapagliflozin group and 286 (12.1%) in the placebo group.

### Patient characteristics

*Table* *1* shows baseline characteristics of those with and without COPD. Patients with COPD were more often male, older and current or ex‐smokers, compared to those without COPD. Patients with COPD had lower (worse) median KCCQ‐TSS score and a worse NYHA functional class distribution than those without COPD. A similar proportion of patients with and without COPD had a history of coronary heart disease, but patients with COPD had worse renal function, a higher prevalence of atrial fibrillation and higher median NT‐proBNP than those without COPD. Patients with COPD were only slightly less likely to be treated with beta‐blocker but were also less likely to be prescribed MRA. COPD patients treated with beta‐blocker were less likely to be taking a non‐selective antagonist and more likely to have been prescribed beta‐1 adrenoceptor selective agent than those without COPD.

**Table 1:** Baseline characteristics: all patients and patients with and without chronic obstructive pulmonary disease

	Total(*n* = 4744)	Without COPD(*n* = 4159)	With COPD(*n* = 585)	*P*‐value
Age (years)	66.3 ± 10.9	66.0 ± 11.0	69.1 ± 9.4	<0.001
Women	1109 (23.4)	1002 (24.1)	107 (18.3)	0.002
Region				<0.001
Asia/Pacific	1096 (23.1)	1010 (24.3)	86 (14.7)	
Europe	2154 (45.4)	1858 (44.7)	296 (50.6)	
North America	677 (14.3)	541 (13.0)	136 (23.2)	
South America	817 (17.2)	750 (18.0)	67 (11.5)	
Race				<0.001
White	3333 (70.3)	2864 (68.9)	469 (80.2)	
Black	226 (4.8)	202 (4.9)	24 (4.1)	
Asian	1116 (23.5)	1028 (24.7)	88 (15.0)	
Other	69 (1.5)	65 (1.6)	4 (0.7)	
HR (bpm)	71.5 ± 11.7	71.4 ± 11.7	72.0 ± 11.4	0.24
SBP (mmHg)	121.8 ± 16.3	121.5 ± 16.3	123.8 ± 16.6	0.001
DBP (mmHg)	73.5 ± 10.5	73.5 ± 10.5	73.2 ± 10.2	0.42
BMI (kg/m^2^)	28.2 ± 6.0	28.1 ± 5.9	28.3 ± 6.4	0.50
Hypertension	3523 (74.3)	3049 (73.3)	474 (81.0)	<0.001
Diabetes	2139 (45.1)	1861 (44.7)	278 (47.5)	0.21
Myocardial infarction	2092 (44.1)	1825 (43.9)	267 (45.6)	0.42
Atrial fibrillation	1818 (38.3)	1557 (37.4)	261 (44.6)	<0.001
Stroke	466 (9.8)	405 (9.7)	61 (10.4)	0.60
HF aetiology				0.49
Ischaemic	2674 (56.4)	2331 (56.0)	343 (58.6)	
Non‐ischaemic	1687 (35.6)	1489 (35.8)	198 (33.8)	
Unknown	383 (8.1)	339 (8.2)	44 (7.5)	
Previous HF hospitalization	2251 (47.4)	1951 (46.9)	300 (51.3)	0.047
Smoking status				<0.001
Never	1959 (41.3)	1854 (44.6)	105 (18.0)	
Former	2092 (44.1)	1770 (42.6)	322 (55.0)	
Current	693 (14.6)	535 (12.9)	158 (27.0)	
KCCQ‐TSS	77 (58–92)	79 (60–93)	71 (53–85)	<0.001
NYHA class III/IV	1541 (32.5)	1292 (31.1)	249 (42.6)	<0.001
LVEF (%)	31.1 ± 6.8	31.0 ± 6.8	31.6 ± 6.8	0.036
NT‐proBNP (pg/mL)	1437 (857–2650)	1418 (850–2616)	1574 (893–2807)	0.021
No AF	1254 (744–2341)	1237 (739–2270)	1493 (808–2845)	0.002
With AF	1792 (1107–3056)	1798 (1114–3097)	1704 (1044–2765)	0.18
eGFR (mL/min/1.73 m^2^)	65.8 ± 19.4	66.1 ± 19.4	63.4 ± 19.4	0.001
eGFR <60 mL/min/1.73 m^2^	1926 (40.6)	1652 (39.7)	274 (46.9)	<0.001
Creatinine (µmol/L)	104.4 ± 30.4	104.0 ± 30.0	107.6 ± 32.8	0.007
Haemoglobin (g/L)	135.5 ± 16.2	135.5 ± 16.2	135.8 ± 16.4	0.63
Potassium (mmol/L)	4.5 ± 0.5	4.5 ± 0.5	4.5 ± 0.5	0.60
Diuretic	4433 (93.4)	3885 (93.4)	548 (93.7)	0.81
ACEI	2661 (56.1)	2340 (56.3)	321 (54.9)	0.53
ARB	1307 (27.6)	1161 (27.9)	146 (25.0)	0.13
ARNI	508 (10.7)	437 (10.5)	71 (12.1)	0.23
Beta‐blocker	4558 (96.1)	4018 (96.6)	540 (92.3)	<0.001
≥50% of target dose	2349 (51.5)	2066 (51.4)	283 (52.4)	0.67
Beta‐1 selective^a^	2779 (58.6)	2427 (58.4)	352 (60.2)	0.42
Non‐selective^a^	1775 (37.4)	1587 (38.2)	188 (32.1)	0.005
MRAs	3370 (71.0)	2987 (71.8)	383 (65.5)	0.002
Digoxin	887 (18.7)	786 (18.9)	101 (17.3)	0.34
Ivabradine	228 (4.8)	203 (4.9)	25 (4.3)	0.52
PCI	1624 (34.2)	1415 (34.0)	209 (35.7)	0.42
CABG	799 (16.8)	687 (16.5)	112 (19.1)	0.11
CRT	354 (7.5)	305 (7.3)	49 (8.4)	0.37
ICD	953 (20.1)	830 (20.0)	123 (21.0)	0.55

Data are given as mean ± standard deviation or median (interquartile range) for continuous measures, and *n* (%) for categorical measures.

ACEI, angiotensin‐converting enzyme inhibitor; AF, atrial fibrillation; ARB, angiotensin receptor blocker; ARNI, angiotensin receptor–neprilysin inhibitor; BMI, body mass index; CABG, coronary artery bypass graft; COPD, chronic obstructive pulmonary disease; CRT, cardiac resynchronization therapy; DBP, diastolic blood pressure; eGFR, estimated glomerular filtration rate; HF, heart failure; HR, heart rate; ICD, implantable cardioverter‐defibrillator; KCCQ‐TSS, Kansas City Cardiomyopathy Questionnaire total symptom score; MRA, mineralocorticoid receptor antagonist; NT‐proBNP, N‐terminal pro B‐type natriuretic peptide; NYHA, New York Heart Association; PCI, percutaneous coronary intervention; SBP, systolic blood pressure.

^a^ Four excluded.

Among patients with COPD, 213 (36.4%) were treated with inhaled beta‐agonist, 138 (23.6%) with muscarinic antagonist, and 71 (12.1%) with a corticosteroid (online supplementary *Table* *S1*).

Baseline characteristics were similar in patients with and without COPD randomized to placebo and dapagliflozin (online supplementary *Table* *S1*).

Of the 585 patients with COPD, investigators also reported a diagnosis of asthma in 56 and an additional 133 patients had an investigator‐reported diagnosis of asthma only (online supplementary *Tables* *S2* and *S3*). Patients with asthma only compared to COPD were distinct in several respects, e.g. younger, more likely to be female and much less frequent smoking history. There were also some similarities between patients with COPD and asthma (as compared to patients without COPD/asthma), including worse KCCQ score, lower eGFR and higher prevalence of atrial fibrillation. Although beta‐blocker use was high (91.0%) in patients with asthma only, it was lower than in any other group. Conversely, the use of corticosteroids was highest in patients with asthma, compared with COPD only.

### Hospitalization and mortality outcomes in patients with and without chronic obstructive pulmonary disease

#### Primary outcome

**Figure 1:**
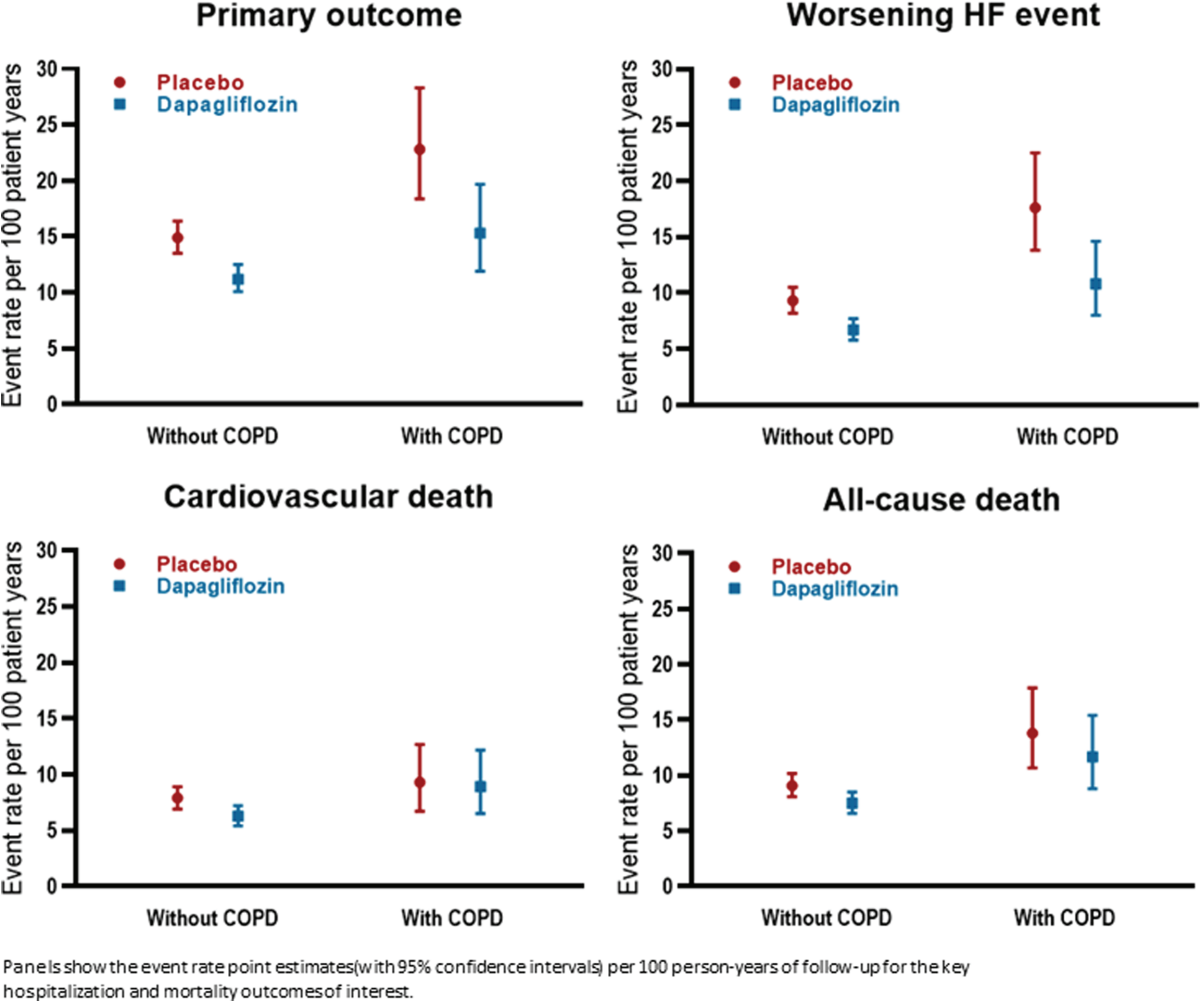
Efficacy of dapagliflozin in DAPA‐HF according to chronic obstructive pulmonary disease (COPD) status at baseline. HF, heart failure.

**Figure 2:**
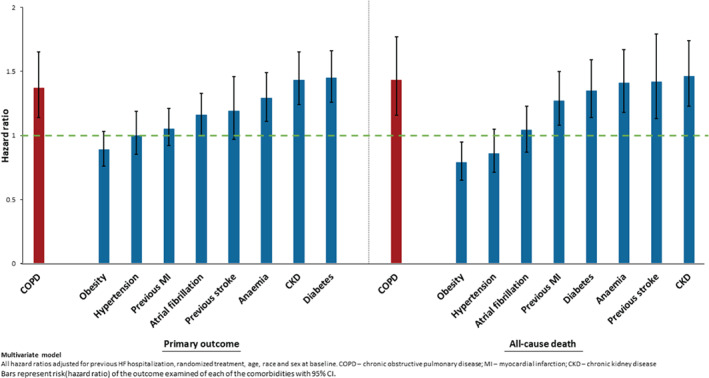
Risk of primary outcome and all‐cause mortality associated with major comorbidities.

The incidence rate (per 100 person‐years) of the primary composite outcome was higher in patients with COPD than in those without [18.9, 95% confidence interval (CI) 16.0–22.2 vs. 13.0, 95% CI 12.1–14.0] (*Table* *2*, *Figure* *1*, *Graphical Abstract* and online supplementary *Figure* *S1*). Elevated risk persisted after adjustment for other prognostic variables and use of a beta‐blocker or MRA. The elevation of risk was somewhat higher when recurrent events were included (*Table* *2*). *Figure* *2* shows the excess risk associated with COPD was similar to the risk associated with chronic kidney disease and diabetes, and greater than the other comorbidities examined.

**Table 2:** Clinical outcomes according to chronic obstructive pulmonary disease status

	Without COPD(*n* = 4159)	With COPD(*n* = 585)
Primary outcome		
Events, *n* (%)	744 (17.9)	144 (24.6)
Event rate/100 person‐ years	13.0 (12.1–14.0)	18.9 (16.0–22.2)
HR	1.00 (ref.)	1.44 (1.21–1.72) <0.001
HR‐1	1.00 (ref.)	1.26 (1.05–1.52) 0.014
HR‐2	1.00 (ref.)	1.24 (1.03–1.50) 0.023
Worsening HF event		
Events, *n* (%)	456 (11.0)	107 (18.3)
Event rate/100 person‐years	8.0 (7.3–8.8)	14.0 (11.6–17.0)
HR	1.00 (ref.)	1.74 (1.41–2.15) <0.001
HR‐1	1.00 (ref.)	1.53 (1.22–1.90) <0.001
HR‐2	1.00 (ref.)	1.51 (1.21–1.89) <0.001
First HF hospitalization		
Events, *n* (%)	443 (10.7)	106 (18.1)
Event rate/100 person‐years	7.7 (7.1–8.5)	13.9 (11.5–16.8)
HR	1.00 (ref.)	1.78 (1.44–2.20) <0.001
HR‐1	1.00 (ref.)	1.58 (1.26–1.97) <0.001
HR‐2	1.00 (ref.)	1.57 (1.25–1.96) <0.001
Urgent visit for HF		
Events, *n* (%)	30 (0.7)	3 (0.5)
Event rate/100 person‐years	0.5 (0.4–0.7)	0.4 (0.1–1.1)
HR	1.00(ref.)	0.75 (0.23–2.45) 0.629
HR‐1	1.00 (ref.)	0.63 (0.19–2.12) 0.453
HR‐2	1.00 (ref.)	0.54 (0.16–1.86) 0.332
CV death		
Events, *n* (%)	424 (10.2)	76 (13.0)
Event rate/100 person‐years	7.1 (6.4–7.8)	9.1 (7.2–11.4)
HR	1.00 (ref.)	1.28 (1.00–1.63) 0.049
HR‐1	1.00 (ref.)	1.10 (0.85–1.42) 0.453
HR‐2	1.00 (ref.)	1.08 (0.84–1.39) 0.553
Total HF hospitalization/CV death		
Events, *n*	1070	239
Event rate/100 person‐years	17.9 (16.8–19.0)	28.8 (25.3–32.7)
RR	1.00 (ref.)	1.59 (1.31–1.93) <0.001
RR‐1	1.00 (ref.)	1.40 (1.15–1.72) 0.001
RR‐2	1.00 (ref.)	1.39 (1.14–1.70) 0.001
Non‐CV death		
Events, *n* (%)	74 (1.8)	31 (5.3)
Event rate/100 person‐years	1.2 (1.0–1.5)	3.7 (2.6–5.2)
HR	1.00 (ref.)	2.99 (1.97–4.56) <0.001
HR‐1	1.00 (ref.)	2.18 (1.38–3.42) 0.001
HR‐2	1.00 (ref.)	2.23 (1.42–3.51) 0.001
All‐cause death		
Events, *n* (%)	498 (12.0)	107 (18.3)
Event rate/100 person‐years	8.3 (7.6–9.1)	12.7 (10.5–15.4)
HR	1.00 (ref.)	1.53 (1.25–1.89) <0.001
HR‐1	1.00 (ref.)	1.27 (1.02–1.58) 0.031
HR‐2	1.00 (ref.)	1.26 (1.01–1.57) 0.041
KCCQ total symptom score (change assessed at 8 months)		
Mean change ± SD	4.8 ± 18.9	4.1 ± 19.8
Difference^a^	−0.67 (−2.52–1.18)	
Proportion with ≥5 increase	55.4	49.0
Proportion with ≥5 decrease	28.3	35.0

COPD, chronic obstructive pulmonary disease; CV, cardiovascular; HF, heart failure; HR, hazard ratio; KCCQ, Kansas City Cardiomyopathy Questionnaire; RR, rate ratio; SD, standard deviation.

Risk and rate ratios adjusted for randomized treatment and previous HF hospitalization at baseline (except non‐CV and all‐cause death) and stratified by diabetes status.

Worsening HF event‐HF hospitalization/urgent visit requiring intravenous therapy for HF.

Model 1 adjusted for age, sex, region, systolic blood pressure, history of atrial fibrillation, New York Heart Association class III/IV, left ventricular ejection fraction, N‐terminal pro B‐type natriuretic peptide (log), estimated glomerular filtration rate and smoking status.

Model 2 adjusted the same as Model 1 and for baseline beta‐blocker and mineralocorticoid receptor antagonist prescription.

^a^ Indicates difference in means between patients with COPD and without COPD.

#### Worsening heart failure events

Adjusted risk of a worsening HF event was also significantly higher in patients with COPD, compared to those without.

#### Mortality

By contrast, the crude incidence of CV death was only slightly higher in patients with COPD and the adjusted risk was not significantly elevated. However, unadjusted and adjusted risk of death from any cause was higher in patients with COPD, because of a substantially elevated (twofold) risk of non‐CV death (*Table* *2*). The excess of non‐CV causes of death in patients with COPD were those attributed to infection and ‘other’ (online supplementary *Figure* *S2*).

Mortality and hospitalization rates for patients with asthma only (compared with COPD only) are shown in online supplementary *Figure* *S3*. Due to the small number of individuals in the latter group (*n* = 133), formal statistical testing was not done although the rate of HF hospitalization seemed to be almost as high in patients with COPD (but mortality was similar to patients without COPD or asthma).

### Symptoms and quality of life assessed using the Kansas City Cardiomyopathy Questionnaire in patients with and without chronic obstructive pulmonary disease

**Figure 3:**
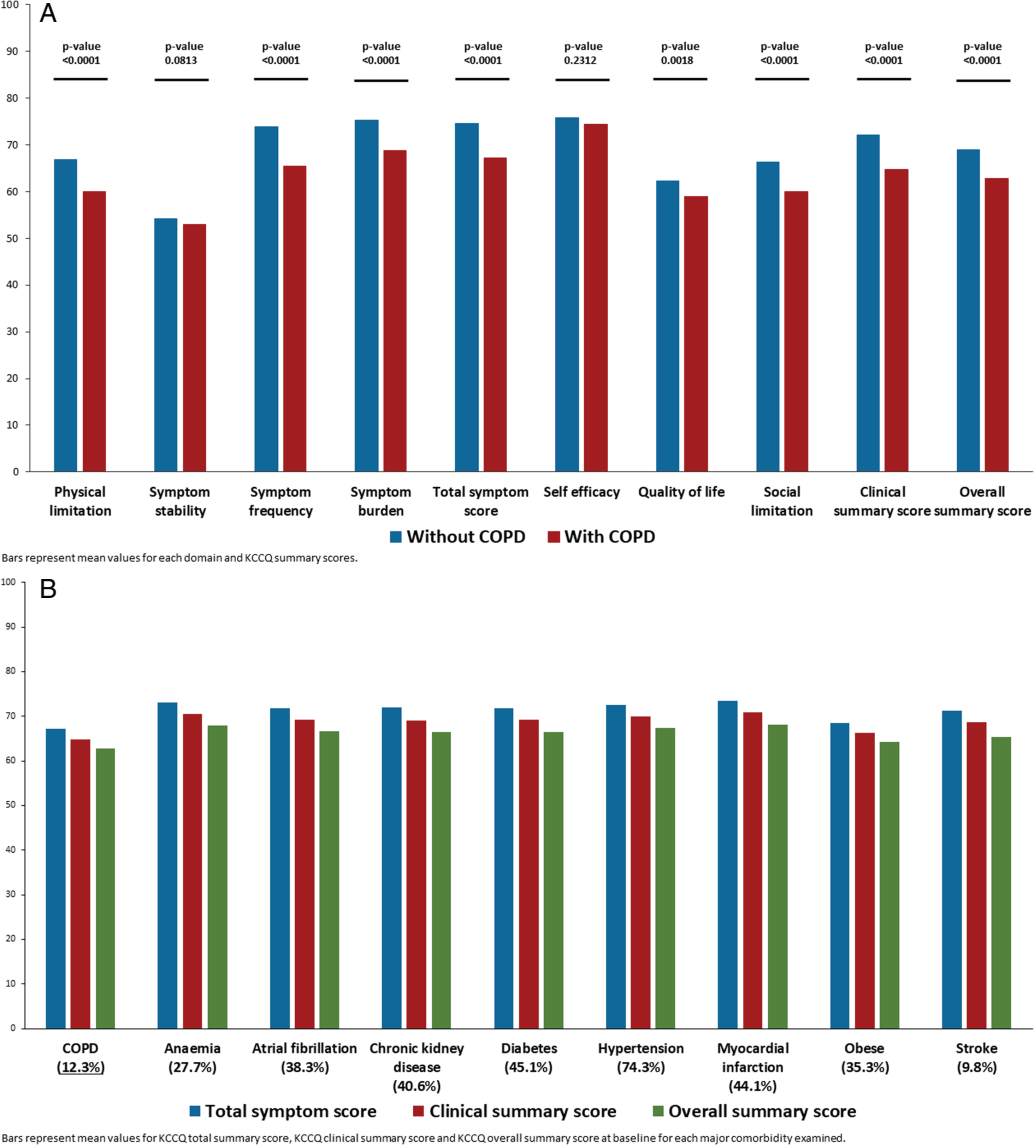
(*A*) Individual Kansas City Cardiomyopathy Questionnaire (KCCQ) domain scores at baseline by chronic obstructive pulmonary disease (COPD) status. (*B*) Baseline KCCQ scores associated with major comorbidities.

*Figure* *3* *A* shows the impact of COPD on self‐reported health status. All but one of the KCCQ domains were significantly worse in patients with COPD, compared to those without. *Figure* *3* *B* shows health status in patients with COPD, compared with other common comorbidities. Each of the KCCQ scores was lower (worse) in patients with COPD than in participants with other comorbidities.

### Effects of dapagliflozin on hospitalization and mortality outcomes

*Table* *3* and *Figure* *1* show the effect of dapagliflozin vs. placebo on pre‐specified outcomes, according to COPD status.

**Table 3:** Clinical outcomes according to randomized treatment in patients with and without chronic obstructive pulmonary disease

	Without COPD		With COPD		Interaction *P*‐value
	Placebo (*n* = 2085)	Dapagliflozin (*n* = 2074)	Placebo (*n* = 286)	Dapagliflozin (*n* = 299)	
Primary outcome					
Events (%)	419 (20.1)	325 (15.7)	83 (29.0)	61 (20.4)	
Event rate/100 person‐years	14.9 (13.5–16.4)	11.2 (10.1–12.5)	22.8 (18.4–28.3)	15.3 (11.9–19.7)	
HR	0.76 (0.65–0.87)		0.67 (0.48–0.93)		0.47
Worsening HF event					
Events (%)	262 (12.6)	194 (9.4)	64 (22.4)	43 (14.4)	
Event rate/100 person‐years	9.3 (8.2–10.5)	6.7 (5.8–7.7)	17.6 (13.8–22.5)	10.8 (8.0–14.6)	
HR	0.72 (0.60–0.87)		0.61 (0.41–0.90)		0.42
First HF hospitalization					
Events (%)	254 (12.2)	189 (9.1)	64 (22.4)	42 (14.1)	
Event rate/100 person‐years	9.0 (8.0–10.2)	6.5 (5.7–7.5)	17.5 (13.7–22.4)	10.5 (7.8–14.3)	
HR	0.73 (0.60–0.88)		0.59 (0.40–0.88)		0.35
CV death					
Events (%)	235 (11.3)	189 (9.1)	38 (13.3)	38 (12.7)	
Event rate/100 person‐years	7.9 (6.9–8.9)	6.3 (5.4–7.2)	9.3 (6.7–12.7)	8.9 (6.5–12.2)	
HR	0.80 (0.66–0.97)		0.96 (0.61–1.51)		0.47
Total HF hospitalization/CV death					
Events	605	465	137	102	
Event rate/100 person‐years	20.3 (18.2–22.7)	15.5 (13.7–17.5)	33.8 (27.0–42.9)	24.0 (18.2–32.1)	
RR	0.76 (0.65–0.90)		0.71 (0.50–1.03)		0.71
KCCQ total symptom score (change assessed at 8 months)					
Mean change ± SD	3.4 ± 19.2	6.2 ± 18.4	2.4 ± 19.2	5.8 ± 20.2	
Between treatment difference	2.73 (1.47–3.99)		3.42 (−0.19–7.04)		0.71
Proportion with ≥5 score increase	51.7	59.2	45.6	52.2	
	1.16 (1.08–1.24)		1.14 (0.96–1.36)		0.87
Proportion with ≥5 score decrease	31.9	24.6	39.9	30.3	
	0.84 (0.78–0.90)		0.81 (0.68–0.96)		0.69
All‐cause death					
Events (%)	272 (13.1)	226 (10.9)	57 (19.9)	50 (16.7)	
Event rate/100 person‐years	9.1 (8.1–10.2)	7.5 (6.6–8.5)	13.8 (10.7–17.9)	11.7 (8.8–15.4)	
HR	0.83 (0.69–0.99)		0.83 (0.57–1.22)		0.96

COPD, chronic obstructive pulmonary disease; CV, cardiovascular; HF, heart failure; HR, hazard ratio; KCCQ, Kansas City Cardiomyopathy Questionnaire; RR, rate ratio; SD, standard deviation.

Risk and rate ratios adjusted for previous HF hospitalization at baseline (except all‐cause death) and stratified by diabetes status.

Worsening HF event‐HF hospitalization/urgent visit requiring intravenous therapy for HF.

#### Primary outcome

The effect of dapagliflozin, compared with placebo, on the primary outcome was consistent in patients with COPD [hazard ratio (HR) 0.67, 95% CI 0.48–0.93] and without COPD (HR 0.76, 95% CI 0.65–0.87; interaction *P*‐value 0.47) (*Table* *3* and *Figure* *1*).

#### Worsening heart failure events

The benefit of dapagliflozin, compared with placebo, on worsening HF events was also consistent in patients with and without COPD (*Table* *3* and *Figure* *1*).

#### Mortality

The effects of dapagliflozin on CV (interaction *P*‐value 0.47) and all‐cause mortality (interaction *P*‐value 0.96) were also consistent in patients with and without COPD (*Table* *3* and *Figure* *1*).

### Effect of dapagliflozin on symptoms and quality of life assessed using the Kansas City Cardiomyopathy Questionnaire

**Figure 4:**
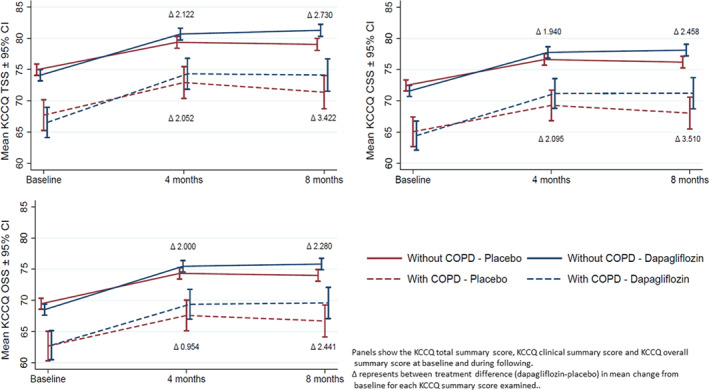
Effect of randomized treatment on change in Kansas City Cardiomyopathy Questionnaire (KCCQ) scores from baseline to 8 months according to chronic obstructive pulmonary disease (COPD) status.

In the pre‐specified KCCQ analysis, the improvement in KCCQ‐TSS with dapagliflozin, compared to placebo, was similar in patients with and without COPD (interaction *P*‐value 0.87) and the same was true for the exploratory analyses of KCCQ‐CSS and KCCQ‐OSS (*Table* *3* and *Figure* *4*).

### Absolute benefits of dapagliflozin in patients with and without chronic obstructive pulmonary disease

Applying the overall relative risk reduction (26%) to the placebo group event rate in those with COPD gave an absolute risk reduction of 5.9 fewer patients experiencing a primary outcome per 100 person‐years. The equivalent reduction in patients without COPD was 3.9 fewer patients per 100 person‐years.

Applying the overall relative risk reduction (30%) to the placebo group event rate in participants with COPD gave an absolute risk reduction of 5.3 fewer patients experiencing a worsening HF event, per 100 person‐years of follow‐up. The equivalent reduction in patients without COPD was 2.8 per 100 person‐years.

The equivalent figures for death from any cause were 2.3 fewer per 100 person‐years patients with COPD and 1.5 fewer per 100 person‐years in patients without COPD.

### Pre‐specified safety assessments

The proportion of patients stopping study drug for any reason in the placebo group was higher in patients with COPD, compared to those without COPD (*Table* *4*). However, the rate of discontinuation was similar between dapagliflozin and placebo in patients with and without COPD (interaction *P*‐value 0.57).

**Table 4:** Pre‐specified safety outcomes and discontinuation according to randomized treatment and chronic obstructive pulmonary disease status^a^

	Without COPD		With COPD		Interaction *P*‐value
	Placebo (*n* = 2083)	Dapagliflozin (*n* = 2071)	Placebo (*n* = 285)	Dapagliflozin (*n* = 297)	
Any study drug discontinuation					
Events (%)	219/2085 (10.5)	214/2074 (10.3)	39/286 (13.6)	35/299 (11.7)	
OR	0.98 (0.80–1.20)		0.84 (0.51–1.38)		0.57
AE related study drug discontinuation					
Events (%)	95 (4.6)	93 (4.5)	21 (7.4)	18 (6.1)	
OR	0.98 (0.73–1.32)		0.80 (0.42–1.54)		0.59
Volume depletion					
Events (%)	140 (6.7)	153 (7.4)	22 (7.7)	25 (8.4)	
OR	1.11 (0.87–1.41)		1.08 (0.59–1.97)		0.96
Renal AE					
Events (%)	137 (6.6)	123 (5.9)	33 (11.6)	30 (10.1)	
OR	0.90 (0.70–1.16)		0.84 (0.50–1.42)		0.81

AE, adverse event; COPD, chronic obstructive pulmonary disease; OR, odds ratio.

OR (95% confidence interval) adjusted for baseline diabetes status.

^a^ Only in safety set except for discontinuation due to any cause.

Adverse events related to volume depletion were reported in 7.7% of the placebo group and in 8.4% in the dapagliflozin group in patients with COPD, compared to 6.7% and 7.4%, respectively, in patients without COPD (*Table* *4* and *Graphical Abstract*). The rate of renal AEs was numerically (but not significantly) lower in patients treated with dapagliflozin, compared with placebo, both in patients with and without COPD (interaction *P*‐value 0.81).

## Discussion

In DAPA‐HF, patients with COPD were older and more commonly men with a history of smoking and atrial fibrillation and had worse renal function and a higher NT‐proBNP level, than participants without COPD. Patients with COPD were slightly less likely to be treated with a beta‐blocker or MRA and had more severe functional limitation and impairment of quality of life than participants without COPD. During follow‐up, patients with COPD experienced higher rates of the primary composite endpoint and key secondary endpoints; fewer had a clinically meaningful improvement (and more deterioration) in symptoms and quality of life, compared to those without COPD. Efficacy and tolerability of dapagliflozin were consistent in participants with and without COPD, with greater absolute risk reductions in hospitalization and death in COPD patients due to their higher overall event rates. Mean improvements in symptoms and quality of life were numerically larger in patients with COPD, compared to those without.

In DAPA‐HF, 12.3% of patients had concomitant COPD, very similar to the frequency reported in most other trials, including PARADIGM‐HF, where prevalence was 12.9%.^4^, ^5^, ^6^, ^19^, ^20^, ^21^ However, this is likely to be lower than the true prevalence of COPD in unselected patients with HFrEF for two reasons. First, the inclusion and exclusion criteria used in trials, including the requirement for patients to be treated with beta‐blocker, unless contraindicated or not tolerated, likely led to under‐enrolment of patients with severe COPD. Second, use of spirometry would likely have detected undiagnosed COPD. However, the prevalence of COPD in recent registry studies has not been much higher. In the European Society of Cardiology HF Long‐Term Registry, the prevalence of COPD was 14.1%.^22^ Moreover, 23% of patients in that registry had HF with preserved ejection fraction (HFpEF) and COPD is more common in HFpEF than HFrEF.^1^, ^2^, ^3^ The proportion of HFrEF patients with COPD in a US registry was 16.5%.^23^ In a large Asian registry, prevalence of COPD was 8.3% (but varied across Asia from 4.7% to 11%).^24^

As expected, patients with COPD in DAPA‐HF had more adverse characteristics including older age and more frequent history of hypertension and, notably, atrial fibrillation. The possibility that beta‐agonists increase the risk of atrial fibrillation has been raised previously.^1^

Although prior and current smoking were, as expected, more common in patients with COPD, coronary heart disease was not more common. We also found patients with COPD had a higher mean NT‐proBNP level, although only modestly so (and this was only the case in patients without atrial fibrillation). There was no clinically relevant difference in LVEF between patients with and without COPD. The latter findings contrasted strikingly with the substantially higher proportion of patients with COPD reported to be in NYHA class III/IV and the significantly lower (worse) KCCQ‐TSS (and other KCCQ scores) in people with COPD compared to those without. Indeed, all but one of the domains of KCCQ was worse in patients with COPD compared to most other comorbidities. Although a similar overall mean decrement (−8 points) in KCCQ‐OSS was reported in the Heart Failure: A Controlled Trial Investigating Outcomes of Exercise Training trial (HF‐ACTION), we do not know of any other description of the impact of COPD across domains of quality of life/health status or any comparison of the impact of COPD compared to other comorbidities.^5^

Interestingly, and in contrast to most prior studies, we found beta‐blocker use was high in patients with COPD (92.3%) although not as high as in patients without COPD (96.6%, *P* < 0.001). This finding may indicate that the recommendation in HF guidelines that COPD is not a contraindication to use of a beta‐blocker may have been heeded in this selected clinical trial population.^5^, ^19^, ^20^, ^25^, ^26^ More surprisingly, however, was the finding that MRA use was also significantly less common in patients with COPD, despite their worse functional class. A likely explanation is the higher prevalence of renal dysfunction among patients with COPD, compared to those without COPD.^27^

Even after adjusting for differences in demographics, comorbidity, key disease‐modifying therapy and NT‐proBNP, COPD remained an independent predictor of the primary outcome, although the impact was greater on worsening HF events that on CV death. However, there was a clear association between COPD and death from any cause because of a higher risk of non‐CV death in patients with COPD. The excess risk associated with COPD was striking when compared with other common comorbidities, with only chronic kidney disease and diabetes showing a similar hazard; we are not aware of any comparative analysis of this type.

These data and our earlier observations on symptoms/quality of life raise two questions about the interaction between COPD and HFrEF. The first is why is COPD associated with worse symptoms and functional status and a higher risk of HF hospitalization? The explanation for the former could simply be that patients experience the extra impact of two cardiac and respiratory conditions causing dyspnoea and effort intolerance (and, potentially, the additional burden of atrial fibrillation). This does not readily explain higher natriuretic peptide levels in patients in sinus rhythm, which may be due to the impact of COPD on pulmonary artery pressure and right ventricular function.^1^, ^7^, ^28^

The benefits of dapagliflozin were consistent in patients with and without COPD, both for worsening HF events and death. This finding is especially important because risk was greater in patients with COPD and, therefore, the absolute risk reduction was larger in these individuals, than in participants without COPD and also because this risk reduction persisted despite the competing (nearly twofold) risk of non‐CV death in the COPD group.

Similarly, dapagliflozin was as well tolerated, compared with placebo, in patients with and without COPD. Collectively, this preserved efficacy and tolerability is very important, given the risk faced by patients with COPD and the more limited alternative options for at least some of these patients.^28^, ^29^

### Limitations

This study has several limitations. The analysis was not pre‐specified and the proportion of patients with COPD was relatively small, compared to those without. COPD was investigator‐reported, and it is likely that the true prevalence of COPD would probably be higher if all patients had performed spirometry. Participants in this study were selected for a randomized controlled trial and were probably healthier, overall, than ‘real‐world’ patients. Investigators were asked not to include patients with another condition likely to lead to a life‐expectancy of <2 years, which may have led to exclusion of patients with severe COPD. The high rate of use of beta‐blockers is also consistent with the trial participants representing healthier, better‐treated, patients enrolled at sites practicing evidence‐based medicine.

## Conclusion

In summary, in DAPA‐HF, approximately one in eight patients with HFrEF had concomitant COPD. Participants with COPD had worse symptoms and functional limitation, compared to those without, and a higher risk of HF hospitalization and death from any cause. The relative risk reduction with dapagliflozin on all pre‐specified mortality/morbidity outcomes was the same in patients with and without COPD (and absolute risk reduction greater in those with COPD because of their higher baseline risk), as was the improvement in symptoms. Dapagliflozin was equally well tolerated, compared with placebo, in patients with and without COPD.

### Funding

The DAPA‐HF trial was funded by AstraZeneca. Prof McMurray is supported by British Heart Foundation Centre of Research Excellence Grant RE/18/6/34217.

**Conflict of interest**: K.F.D. received grant support from Novartis. O.B., A.M.L., D.L. and M.S. are full‐time employees of AstraZeneca. R.A.B. received grant support (paid to University Medical Center Groningen [UMCG]), consulting fees, and lecture fees from AstraZeneca, grant support (paid to UMCG) from Bristol‐Myers Squibb, grant support (paid to UMCG) and consulting fees from Abbott, grant support (paid to UMCG) and lecture fees from Roche, and consulting fees from MandalMed and is a minority shareholder in scPharmaceuticals. A.S.D. received consulting fees from Abbott, Biofourmis, Boston Scientific, Boehringer Ingelheim, DalCor Pharmaceuticals, and Regeneron, grant support (paid to Brigham and Women's Hospital) and consulting fees from Alnylam Pharmaceuticals and Novartis, and advisory board fees from Corvidia and Relypsa. J.D. received personal fees from Berlin Chemie Menarini. S.E.I. reports personal fees and non‐financial support from AstraZeneca, Boehringer Ingelheim, Sanofi/Lexicon, Merck, VTV Therapeutics, and Abbott/Alere, as well as personal fees from AstraZeneca and Zafgen. M.K. reports grant support and lecture fees from Astellas Pharma, Sanofi, Pfizer, Ono Pharmaceutical, Novartis, and Mitsubishi Tanabe Pharma, lecture fees from Daiichi‐Sankyo, Bayer, Boehringer Ingelheim, Kowa Pharmaceutical, Sawai Pharmaceutical, MSD, Shionogi, Kureha, Taisho Toyama Pharmaceutical, Takeda Pharmaceutical, and Toa Eiyo, and manuscript fees from Japan Medical Data Center. L.K. reports other support from AstraZeneca and personal fees from Novartis and Bristol‐Myers Squibb as a speaker. M.N.K. reports personal fees from AstraZeneca; grants, personal fees, and other from AstraZeneca; grants and personal fees from Boehringer Ingelheim; is consultant for Vifor Pharma and personal fees from Sanofi, Amgen, NovoNordisk, Merck (Diabetes), Janssen, Bayer, GlaxoSmithKline, Glytec, Novartis, Applied Therapeutics, Amarin, and Eli Lilly. F.A.M. reports personal fees from AstraZeneca. B.M. reports lecture fees from AstraZeneca, Sanofi Aventis, Servier, and Biotronik and grant support and lecture fees from Abbott and Medtronic. M.C.P. received lecture fees from AstraZeneca, Novartis, and Eli Lilly, grant support, advisory board fees, and fees for serving on an end‐point committee from Boehringer Ingelheim, advisory board fees, lecture fees, and fees for serving on an end‐point committee from Novo Nordisk, advisory board fees from Napp Pharmaceuticals, and fees for serving on an end‐point committee from Takeda Pharmaceutical and Bayer. P.P. reports personal fees and other from AstraZeneca, Boehringer Ingelheim, Bayer, BMS, Cibiem, Novartis, and RenalGuard; personal fees from Pfizer, Servier, Respicardia, and Berlin‐Chemie; other from Amgen; and grants, personal fees, and other from Vifor Pharma. M.S.S. reports grants from Bayer, Daiichi‐Sankyo, Eisai, GlaxoSmithKline, Pfizer, Poxel, Quark Pharmaceuticals, and Takeda; grants and personal fees from Amgen, AstraZeneca, Intarcia, Janssen Research and Development, The Medicines Company, MedImmune, Merck, and Novartis; and personal fees from Anthos Therapeutics, Bristol‐Myers Squibb, CVS Caremark, DalCor, Dyrnamix, Esperion, IFM Therapeutics, and Ionis. Dr Sabatine is a member of the TIMI Study Group, which has also received institutional research grant support through Brigham and Women's Hospital from Abbott, Aralez, Roche, and Zora Biosciences. M.S. reported receiving personal fees and non‐financial support from AstraZeneca and personal fees from Novo Nordisk and Boehringer Ingelheim outside the submitted work. S.D.S. reports grants from AstraZeneca, Bellerophon, Celladon, Ionis, Lone Star Heart, Mesoblast, National Institutes of Health/National Heart, Lung, and Blood Institute, Sanofi Pasteur, and Eidos; grants and personal fees from Alnylam, Amgen, AstraZeneca, BMS, Gilead, GSK, MyoKardia, Novartis, Theracos, Bayer, and Cytokinetics; and personal fees from Akros, Corvia, Ironwood, Merck, Roche, Takeda, Quantum Genomics, AoBiome, Janssen, Cardiac Dimensions, Tenaya, and Daichi‐Sankyo. S.V. received grant support, lecture fees, and advisory board fees from AstraZeneca, Boehringer Ingelheim, Bayer, Janssen, and Merck, lecture fees from Sun Pharmaceutical Industries and EOCI Pharmacomm, grant support and advisory board fees from Amgen, and lecture fees and advisory board fees from Sanofi and Eli Lilly. P.S.J. reports other from AstraZeneca, personal fees from Novartis and Cytokinetics, and grants from Boehringer Ingelheim. J.J.V.M. reports non‐financial support and other from AstraZeneca, Cardiorentis, Amgen, Oxford University/Bayer, Theracos, Abbvie, Novartis, Glaxo Smith Kline, Vifor‐Fresenius, Kidney Research UK, and Novartis, as well as other support from Bayer, DalCor, Pfizer, Merck, Bristol Myers, and Squibb.

## Supporting information


**Figure S1** **.** Clinical outcomes in HFrEF according to chronic obstructive pulmonary disease status at baseline.
**Figure S2.** Causes of death according to chronic obstructive pulmonary disease status.
**Figure S3.** Clinical outcomes according to chronic obstructive pulmonary disease and asthma status.
**Table S1.** Baseline characteristics by treatment and chronic obstructive pulmonary disease status.
**Table S2.** Baseline characteristics by asthma status.
**Table S3.** Baseline characteristics in patients with chronic obstructive pulmonary disease ± asthma.
