# Supplementary material for: Effects of dapagliflozin in heart failure with reduced ejection fraction and chronic obstructive pulmonary disease: an analysis of DAPA‐HF
Source: Eur J Heart Fail. 2021 Jan 18;23(4):632–43. doi: 10.1002/ejhf.2083 (PMC8247863; doi:10.1002/ejhf.2083)
Supplement: Supplementary file 1 — Figure S1 . Clinical outcomes in HFrEF according to chronic obstructive pulmonary disease status at baseline. Figure S2. Causes of death according to chronic obstructive pulmonary disease status. Figure S3. Clinical outcomes according to chronic obstructive pulmonary disease and asthma status. Table S1. Baseline characteristics by treatment and chronic obstructive pulmonary disease status. Table S2. Baseline characteristics by asthma status. Table S3. Baseline characteristics in patients with chronic obstructive pulmonary disease ± asthma. [file EJHF-23-632-s001.docx]

**SUPPLEMENTARY APPENDIX**

**ONLINE FIGURE LEGEND**

**Online Figure 1:**

Clinical outcomes in HFrEF according to COPD status at baseline.

**Online Figure 2:**

Causes of death according to COPD status.

**Online Figure 3:**

Clinical outcomes according to COPD and asthma status

| **Online Table 1: Baseline characteristics by treatment and chronic obstructive pulmonary disease (COPD) status** | | | | |
| --- | --- | --- | --- | --- |
|  | **Without COPD** | | **With COPD** | |
|  | **Placebo**  **(N=2085)** | **Dapagliflozin**  **(N=2074)** | **Placebo**  **(N=286)** | **Dapagliflozin**  **(N=299)** |
| Age (years) | 66.1 ± 10.9 | 65.8 ± 11.1 | 69.2 ± 9.5 | 69.0 ± 9.4 |
| Female sex | 492 (23.6) | 510 (24.6) | 53 (18.5) | 54 (18.1) |
| Region |  |  |  |  |
| Asia/Pacific | 511 (24.5) | 499 (24.1) | 42 (14.7) | 44 (14.7) |
| Europe | 916 (43.9) | 942 (45.4) | 144 (50.3) | 152 (50.8) |
| North America | 275 (13.2) | 266 (12.8) | 67 (23.4) | 69 (23.1) |
| South America | 383 (18.4) | 367 (17.7) | 33 (11.5) | 34 (11.4) |
| Race |  |  |  |  |
| White | 1442 (69.2) | 1422 (68.6) | 229 (80.1) | 240 (80.3) |
| Black | 92 (4.4) | 110 (5.3) | 12 (4.2) | 12 (4.0) |
| Asian | 521 (25.0) | 507 (24.4) | 43 (15.0) | 45 (15.1) |
| Other | 30 (1.4) | 35 (1.7) | 2 (0.7) | 2 (0.7) |
| HR (bpm) | 71.4 ± 11.8 | 71.4 ± 11.7 | 72.1 ± 11.6 | 72.0 ± 11.2 |
| SBP (mmHg) | 121.3 ± 16.2 | 121.8 ± 16.4 | 124.0 ± 17.4 | 123.7 ± 15.8 |
| DBP (mmHg) | 73.4 ± 10.5 | 73.7 ± 10.5 | 73.0 ± 10.2 | 73.3 ± 10.2 |
| BMI (kg/m2) | 28.1 ± 5.9 | 28.1 ± 5.9 | 28.2 ± 6.6 | 28.4 ± 6.3 |
| Hypertension | 1532 (73.5) | 1517 (73.1) | 229 (80.1) | 245 (81.9) |
| Diabetes | 934 (44.8) | 927 (44.7) | 130 (45.5) | 148 (49.5) |
| Myocardial infarction | 933 (44.7) | 892 (43.0) | 132 (46.2) | 135 (45.2) |
| Atrial fibrillation | 779 (37.4) | 778 (37.5) | 123 (43.0) | 138 (46.2) |
| Stroke | 212 (10.2) | 193 (9.3) | 28 (9.8) | 33 (11.0) |
| HF aetiology |  |  |  |  |
| Ischaemic | 1185 (56.8) | 1146 (55.3) | 173 (60.5) | 170 (56.9) |
| Non-Ischaemic | 742 (35.6) | 747 (36.0) | 88 (30.8) | 110 (36.8) |
| Unknown | 158 (7.6) | 181 (8.7) | 25 (8.7) | 19 (6.4) |
| Previous HF hospitalization | 975 (46.8) | 976 (47.1) | 152 (53.1) | 148 (49.5) |
| KCCQ TSS | 79 (61 - 94) | 79 (59 - 93) | 70 (54 - 84) | 71 (51 - 88) |
| NYHA III/IV | 651 (31.2) | 641 (30.9) | 123 (43.0) | 126 (42.1) |
| LVEF (%) | 30.8 ± 6.9 | 31.2 ± 6.7 | 31.7 ± 6.5 | 31.5 ± 7.0 |
| NT-proBNP (pg/ml) | 1430 (846 - 2615) | 1409 (859 - 2616) | 1579 (931 - 2765) | 1562 (850 - 2841) |
| No AFib | 1241 (737 - 2257) | 1229 (742 - 2311) | 1423 (857 - 2776) | 1526 (768 - 2885) |
| With AFib | 1809 (1114 - 3229) | 1784 (1113 - 2984) | 1837 (1136 - 2765) | 1587 (1006 - 2787) |
| eGFR (ml/min/1.73m^2^) | 65.9 ± 19.2 | 66.3 ± 19.5 | 62.7 ± 19.2 | 64.0 ± 19.6 |
| eGFR<60 | 829 (39.8) | 823 (39.7) | 135 (47.2) | 139 (46.6) |
| Creatinine (μmol/l) | 104.3 ± 30.4 | 103.7 ± 29.7 | 109.2 ± 35.3 | 106.1 ± 30.2 |
| Haemoglobin (g/l) | 135.6 ± 15.9 | 135.3 ± 16.4 | 136.6 ± 15.8 | 135.1 ± 16.9 |
| Potassium(nmol/l) | 4.5 ± 0.5 | 4.5 ± 0.5 | 4.6 ± 0.6 | 4.5 ± 0.5 |
| Diuretics | 1950 (93.5) | 1935 (93.3) | 267 (93.4) | 281 (94.0) |
| ACEI | 1170 (56.1) | 1170 (56.4) | 159 (55.6) | 162 (54.2) |
| ARB | 563 (27.0) | 598 (28.8) | 69 (24.1) | 77 (25.8) |
| ARNI | 225 (10.8) | 212 (10.2) | 33 (11.5) | 38 (12.7) |
| Beta-blocker | 2016 (96.7) | 2002 (96.5) | 264 (92.3) | 276 (92.3) |
| ≥50 % of target dose | 1030 (51.1) | 1036 (51.7) | 140 (53.0) | 143 (51.8) |
| Beta-1 selective* | 1203 (57.8) | 1215 (58.6) | 177 (61.9) | 174 (58.2) |
| Non-selective* | 806 (38.7) | 781 (37.7) | 86 (30.1) | 102 (34.1) |
| MRAs | 1494 (71.7) | 1493 (72.0) | 180 (62.9) | 203 (67.9) |
| Digoxin | 394 (18.9) | 392 (18.9) | 48 (16.8) | 53 (17.7) |
| Ivabradine | 96 (4.6) | 107 (5.2) | 13 (4.5) | 12 (4.0) |
| PCI | 713 (34.2) | 702 (33.8) | 109 (38.1) | 100 (33.4) |
| CABG | 352 (16.9) | 335 (16.2) | 63 (22.0) | 49 (16.4) |
| CRT | 141 (6.8) | 164 (7.9) | 23 (8.0) | 26 (8.7) |
| ICD | 425 (20.4) | 405 (19.5) | 61 (21.3) | 62 (20.7) |
| Respiratory system drugs |  |  |  |  |
| Adrenergic agonists † | 30 (1.4) | 43 (2.1) | 77 (26.9) | 68 (22.7) |
| Adrenergic agonists (in combinations) † ‡ | 26 (1.2) | 29 (1.4) | 67 (23.4) | 52 (17.4) |
| Any inhaled adrenergic agonist † | 50 (2.4) | 66 (3.2) | 118 (41.3) | 95 (31.8) |
| Muscarinic antagonists † | 14 (0.7) | 20 (1.0) | 78 (27.3) | 60 (20.1) |
| Glucocorticoids† | 17 (0.8) | 28 (1.4) | 37 (12.9) | 34 (11.4) |
| Systemic adrenergic agonists | 1 (0.0) | 0 (0.0) | 5 (1.7) | 4 (1.3) |
| Other drugs | 3 (0.1) | 1 (0.1) | 21 (7.3) | 10 (3.3) |
| Diabetes medications^§^ |  |  |  |  |
| Biguanides | 454 (51.7) | 436 (50.8) | 58 (51.8) | 68 (50.7) |
| DPP-4 inhibitors | 135 (15.4) | 146 (17.0) | 14 (12.5) | 15 (11.2) |
| GLP-1 analogues | 9 (1.0) | 7 (0.8) | 1 (0.9) | 4 (3.0) |
| Sulfonylureas | 186 (21.2) | 194 (22.6) | 24 (21.4) | 34 (25.4) |
| Insulin | 229 (26.1) | 242 (28.2) | 37 (33.0) | 32 (23.9) |
| Data are presented as mean ± SD or median (IQR) for continuous measures and n (%) for categorical measures.  HR – heart rate bpm – beats per minute SBP – systolic blood pressure BMI – body mass index HF – heart failure KCCQ TSS – Kansas city cardiomyopathy questionnaire total symptom score NYHA – New York heart association classification NT-proBNP – N terminal pro B-type natriuretic peptide eGFR – estimated glomerular filtration rate ACEI – angiotensin converting enzyme inhibitor ARB – angiotensin receptor blocker ARNI – angiotensin receptor blocker-neprilysin inhibitor PCI – primary coronary intervention CABG – coronary bypass surgery CRT – cardiac resynchronization therapy ICD – implantable cardioverter defibrillator.  *4 patients excluded  †Inhaled.  ^‡^ In combination with corticosteroids /antimuscarinics/ other drugs.  ^§^Only in patients with a medical history of diabetes (1983) | | | | |

| **Online Table 2: Baseline characteristics by asthma status.** | | | | |
| --- | --- | --- | --- | --- |
|  | **Total** | **Without asthma** | **With asthma** | **p-value** |
|  | **(N=4744)** | **(N=4555)** | **(N=189)** |  |
| Age (years) | 66.3 ± 10.9 | 66.3 ± 10.9 | 67.9 ± 10.0 | 0.046 |
| Female sex | 1109 (23.4) | 1043 (22.9) | 66 (34.9) | <0.001 |
| Region |  |  |  | <0.001 |
| Asia/Pacific | 1096 (23.1) | 1056 (23.2) | 40 (21.2) |  |
| Europe | 2154 (45.4) | 2074 (45.5) | 80 (42.3) |  |
| North America | 677 (14.3) | 629 (13.8) | 48 (25.4) |  |
| South America | 817 (17.2) | 796 (17.5) | 21 (11.1) |  |
| Race |  |  |  | 0.007 |
| White | 3333 (70.3) | 3207 (70.4) | 126 (66.7) |  |
| Black | 226 (4.8) | 209 (4.6) | 17 (9.0) |  |
| Asian | 1116 (23.5) | 1076 (23.6) | 40 (21.2) |  |
| Other | 69 (1.5) | 63 (1.4) | 6 (3.2) |  |
| HR (bpm) | 71.5 ± 11.7 | 71.4 ± 11.6 | 73.3 ± 12.7 | 0.033 |
| SBP (mmHg) | 121.8 ± 16.3 | 121.9 ± 16.3 | 120.3 ± 15.7 | 0.20 |
| DBP (mmHg) | 73.5 ± 10.5 | 73.6 ± 10.5 | 71.7 ± 9.6 | 0.016 |
| BMI (kg/m^2^) | 28.2 ± 6.0 | 28.1 ± 5.9 | 30.0 ± 7.6 | <0.001 |
| Hypertension | 3523 (74.3) | 3381 (74.2) | 142 (75.1) | 0.78 |
| Diabetes | 2139 (45.1) | 2048 (45.0) | 91 (48.1) | 0.39 |
| Myocardial infarction | 2092 (44.1) | 2028 (44.5) | 64 (33.9) | 0.004 |
| Atrial fibrillation | 1818 (38.3) | 1739 (38.2) | 79 (41.8) | 0.32 |
| Stroke | 466 (9.8) | 449 (9.9) | 17 (9.0) | 0.70 |
| HF aetiology |  |  |  | <0.001 |
| Ischaemic | 2674 (56.4) | 2596 (57.0) | 78 (41.3) |  |
| Non-Ischaemic | 1687 (35.6) | 1596 (35.0) | 91 (48.1) |  |
| Unknown | 383 (8.1) | 363 (8.0) | 20 (10.6) |  |
| Previous HF hospitalization | 2251 (47.4) | 2164 (47.5) | 87 (46.0) | 0.69 |
| Smoking Status |  |  |  | 0.25 |
| Never | 1959 (41.3) | 1870 (41.1) | 89 (47.1) |  |
| Former | 2092 (44.1) | 2016 (44.3) | 76 (40.2) |  |
| Current | 693 (14.6) | 669 (14.7) | 24 (12.7) |  |
| KCCQ TSS | 77 (58 - 92) | 78 (58 - 92) | 73 (54 - 88) | 0.029 |
| NYHA III/IV | 1541 (32.5) | 1476 (32.4) | 65 (34.4) | 0.57 |
| LVEF (%) | 31.1 ± 6.8 | 31.1 ± 6.8 | 30.1 ± 6.8 | 0.044 |
| NT-proBNP (pg/ml) | 1437.4 (856.8 - 2649.6) | 1434.0 (854.0 - 2641.1) | 1504.3 (981.0 - 2900.1) | 0.42 |
| eGFR (ml/min/1.73m2) | 65.8 ± 19.4 | 66.0 ± 19.4 | 61.3 ± 18.9 | 0.001 |
| Creatinine (μmol/l) | 104.4 ± 30.4 | 104.3 ± 30.3 | 108.2 ± 32.4 | 0.080 |
| Baseline Potassium (nmol/L) | 4.5 ± 0.5 | 4.5 ± 0.5 | 4.4 ± 0.5 | 0.001 |
| Haemoglobin (g/l) | 135.5 ± 16.2 | 135.6 ± 16.2 | 133.9 ± 15.9 | 0.17 |
| Diuretics | 4433 (93.4) | 4252 (93.3) | 181 (95.8) | 0.19 |
| ACEI | 2661 (56.1) | 2579 (56.6) | 82 (43.4) | <0.001 |
| ARB | 1307 (27.6) | 1257 (27.6) | 50 (26.5) | 0.73 |
| ARNI | 508 (10.7) | 469 (10.3) | 39 (20.6) | <0.001 |
| Beta-blocker | 4558 (96.1) | 4384 (96.2) | 174 (92.1) | 0.004 |
| ≥50 % of target dose | 2349 (51.5) | 2260 (51.6) | 119 (63.0) | 0.013 |
| Beta-1 selective^*^ | 2779 (58.6) | 2666 (58.6) | 113 (59.8) | 0.74 |
| Non-selective^*^ | 1775 (37.4) | 1714 (37.7) | 61 (32.3) | 0.13 |
| MRAs | 3370 (71.0) | 3251 (71.4) | 119 (63.0) | 0.013 |
| Digoxin | 887 (18.7) | 845 (18.6) | 42 (22.2) | 0.20 |
| Ivabradine | 228 (4.8) | 213 (4.7) | 15 (7.9) | 0.040 |
| PCI | 1624 (34.2) | 1567 (34.4) | 57 (30.2) | 0.23 |
| CABG | 799 (16.8) | 776 (17.0) | 23 (12.2) | 0.080 |
| CRT | 354 (7.5) | 330 (7.2) | 24 (12.7) | 0.005 |
| ICD | 953 (20.1) | 905 (19.9) | 48 (25.4) | 0.063 |
| Respiratory system drugs |  |  |  |  |
| Adrenergic agonists^†^ | 218 (4.6) | 151 (3.3) | 67 (35.4) | <0.001 |
| Adrenergic agonists (in combinations) ^†‡^ | 174 (3.7) | 129 (2.8) | 45 (23.8) | <0.001 |
| Any inhaled adrenergic agonist^†^ | 329 (6.9) | 234 (5.1) | 95 (50.3) | <0.001 |
| Muscarinic antagonists^†^ | 172 (3.6) | 143 (3.1) | 29 (15.3) | <0.001 |
| Glucocorticoids^†^ | 116 (2.4) | 76 (1.7) | 40 (21.2) | <0.001 |
| Systemic adrenergic agonists | 10 (0.2) | 10 (0.2) | 0 (0.0) | 0.52 |
| Other drugs | 35 (0.7) | 32 (0.7) | 3 (1.6) | 0.16 |
| Diabetes medications^§^ |  |  |  |  |
| Biguanides | 1016 (51.2) | 975 (51.4) | 41 (48.2) | 0.572 |
| DPP-4 inhibitors | 310 (15.6) | 297 (15.7) | 13 (15.3) | 0.930 |
| GLP-1 analogues | 21 (1.1) | 20 (1.1) | 1 (1.2) | 0.914 |
| Sulfonylureas | 438 (22.1) | 421 (22.2) | 17 (20.0) | 0.635 |
| Insulin | 540 (27.2) | 517 (27.2) | 85 (27.1) | 0.971 |
| Data are presented as mean ± SD or median (IQR) for continuous measures and n (%) for categorical measures.  HR – heart rate; bpm – beats per minute; SBP – systolic blood pressure; BMI – body mass index; HF – heart failure; KCCQ TSS – Kansas city cardiomyopathy questionnaire; total symptom score; CSS – clinical summary score; OSS – overall summary score; NYHA – New York heart association classification; NT-proBNP – N terminal pro B-type natriuretic peptide; eGFR – estimated glomerular filtration rate; ACEI – angiotensin converting enzyme inhibitor; ARB – angiotensin receptor blocker; ARNI – angiotensin receptor blocker-neprilysin inhibitor; PCI – primary coronary intervention; CABG – coronary bypass surgery; CRT – cardiac resynchronization therapy; ICD – implantable cardioverter defibrillator.  *4 patients excluded  †Inhaled.  ^‡^ In combination with corticosteroids /antimuscarinics/ other drugs.  ^§^Only in patients with a medical history of diabetes (1983). | | | | |

| **Online Table 3: Baseline characteristics in patients with COPD ± asthma.** | | | | | |
| --- | --- | --- | --- | --- | --- |
|  | **Without COPD/asthma** | **COPD only** | **Asthma only** | **COPD & asthma** | **p-value** |
|  | **(N=4026)** | **(N=529)** | **(N=133)** | **(N=56)** |  |
| Age (years) | 65.9 ± 11.0 | 68.9 ± 9.5 | 66.6 ± 10.2 | 70.9 ± 8.9 | <0.001 |
| Female sex | 952 (23.6) | 91 (17.2) | 50 (37.6) | 16 (28.6) | <0.001 |
| Region |  |  |  |  | <0.001 |
| Asia/Pacific | 981 (24.4) | 75 (14.2) | 29 (21.8) | 11 (19.6) |  |
| Europe | 1796 (44.6) | 278 (52.6) | 62 (46.6) | 18 (32.1) |  |
| North America | 515 (12.8) | 114 (21.6) | 26 (19.5) | 22 (39.3) |  |
| South America | 734 (18.2) | 62 (11.7) | 16 (12.0) | 5 (8.9) |  |
| Race |  |  |  |  | <0.001 |
| White | 2777 (69.0) | 430 (81.3) | 87 (65.4) | 39 (69.6) |  |
| Black | 191 (4.7) | 18 (3.4) | 11 (8.3) | 6 (10.7) |  |
| Asian | 999 (24.8) | 77 (14.6) | 29 (21.8) | 11 (19.6) |  |
| Other | 59 (1.5) | 4 (0.8) | 6 (4.5) | 0 (0.0) |  |
| HR (bpm) | 71.4 ± 11.7 | 72.0 ± 11.4 | 73.5 ± 13.3 | 72.7 ± 11.3 | 0.11 |
| SBP (mmHg) | 121.6 ± 16.3 | 124.0 ± 16.6 | 119.6 ± 15.4 | 122.1 ± 16.3 | 0.005 |
| DBP (mmHg) | 73.6 ± 10.5 | 73.5 ± 10.2 | 72.5 ± 9.5 | 69.9 ± 9.7 | 0.040 |
| BMI (kg/m^2^) | 28.0 ± 5.8 | 28.3 ± 6.3 | 30.7 ± 7.7 | 28.4 ± 7.2 | <0.001 |
| Hypertension | 2952 (73.3) | 429 (81.1) | 97 (72.9) | 45 (80.4) | 0.001 |
| Diabetes | 1796 (44.6) | 252 (47.6) | 65 (48.9) | 26 (46.4) | 0.46 |
| Myocardial infarction | 1784 (44.3) | 244 (46.1) | 41 (30.8) | 23 (41.1) | 0.014 |
| Atrial fibrillation | 1501 (37.3) | 238 (45.0) | 56 (42.1) | 23 (41.1) | 0.005 |
| Stroke | 392 (9.7) | 57 (10.8) | 13 (9.8) | 4 (7.1) | 0.79 |
| HF aetiology |  |  |  |  | 0.001 |
| Ischaemic | 2275 (56.5) | 321 (60.7) | 56 (42.1) | 22 (39.3) |  |
| Non-Ischaemic | 1425 (35.4) | 171 (32.3) | 64 (48.1) | 27 (48.2) |  |
| Unknown | 326 (8.1) | 37 (7.0) | 13 (9.8) | 7 (12.5) |  |
| Previous HF hospitalization | 1891 (47.0) | 273 (51.6) | 60 (45.1) | 27 (48.2) | 0.23 |
| Smoking Status |  |  |  |  | <0.001 |
| Never | 1782 (44.3) | 88 (16.6) | 72 (54.1) | 17 (30.4) |  |
| Former | 1726 (42.9) | 290 (54.8) | 44 (33.1) | 32 (57.1) |  |
| Current | 518 (12.9) | 151 (28.5) | 17 (12.8) | 7 (12.5) |  |
| KCCQ TSS | 79 (60 - 93) | 71 (52 - 85) | 73 (52 - 88) | 72 (58 - 86) | <0.001 |
| KCCQ CSS | 75 (58 - 89) | 67 (51 - 82) | 71 (47 - 85) | 69 (53 - 82) | <0.001 |
| KCCQ OSS | 72 (55 - 86) | 65 (48 - 79) | 68 (49 - 85) | 68 (50 - 81) | <0.001 |
| NYHA III/IV | 1246 (30.9) | 230 (43.5) | 46 (34.6) | 19 (33.9) | <0.001 |
| LVEF (%) | 31.0 ± 6.8 | 31.8 ± 6.8 | 30.1 ± 7.0 | 29.9 ± 6.1 | 0.017 |
| NT-proBNP (pg/ml) | 1417.0 (847.5 - 2613.7) | 1572.1 (893.0 - 2793.4) | 1456.0 (987.0 - 2873.9) | 1669.2 (879.4 - 2931.7) | 0.13 |
| eGFR (ml/min/1.73m2) | 66.3 ± 19.4 | 63.6 ± 19.3 | 61.4 ± 18.4 | 61.1 ± 20.3 | <0.001 |
| Creatinine (μmol/l) | 103.9 ± 30.0 | 107.4 ± 32.3 | 107.6 ± 30.4 | 109.6 ± 37.0 | 0.024 |
| Baseline Potassium (nmol/L) | 4.5 ± 0.5 | 4.5 ± 0.5 | 4.4 ± 0.4 | 4.4 ± 0.5 | 0.012 |
| Haemoglobin (g/l) | 135.5 ± 16.2 | 136.3 ± 16.4 | 134.9 ± 15.8 | 131.8 ± 15.8 | 0.23 |
| Diuretics | 3757 (93.3) | 495 (93.6) | 128 (96.2) | 53 (94.6) | 0.58 |
| ACEI | 2281 (56.7) | 298 (56.3) | 59 (44.4) | 23 (41.1) | 0.004 |
| ARB | 1125 (27.9) | 132 (25.0) | 36 (27.1) | 14 (25.0) | 0.51 |
| ARNI | 411 (10.2) | 58 (11.0) | 26 (19.5) | 13 (23.2) | <0.001 |
| Beta-blocker | 3897 (96.8) | 487 (92.1) | 121 (91.0) | 53 (94.6) | <0.001 |
| ≥50 % of target dose | 2002 (51.4) | 258 (53.0) | 64 (52.9) | 25 (47.2) | 0.82 |
| Beta-1 selective^*^ | 2351 (58.5) | 315 (59.5) | 76 (57.1) | 37 (66.1) | 0.65 |
| Non-selective‑^*^ | 1542 (38.3) | 172 (32.5) | 45 (33.8) | 16 (28.6) | 0.023 |
| MRAs | 2901 (72.1) | 350 (66.2) | 86 (64.7) | 33 (58.9) | 0.002 |
| Digoxin | 754 (18.7) | 91 (17.2) | 32 (24.1) | 10 (17.9) | 0.34 |
| Ivabradine | 192 (4.8) | 21 (4.0) | 11 (8.3) | 4 (7.1) | 0.17 |
| PCI | 1378 (34.2) | 189 (35.7) | 37 (27.8) | 20 (35.7) | 0.39 |
| CABG | 672 (16.7) | 104 (19.7) | 15 (11.3) | 8 (14.3) | 0.099 |
| CRT | 288 (7.2) | 42 (7.9) | 17 (12.8) | 7 (12.5) | 0.041 |
| ICD | 797 (19.8) | 108 (20.4) | 33 (24.8) | 15 (26.8) | 0.30 |
| Respiratory system drugs |  |  |  |  |  |
| Adrenergic agonists^†^ | 31 (0.8) | 120 (22.7) | 42 (31.6) | 25 (44.6) | <0.001 |
| Adrenergic agonists (in combinations) ^†‡^ | 28 (0.7) | 101 (19.1) | 27 (20.3) | 18 (32.1) | <0.001 |
| Any inhaled adrenergic agonist^†^ | 53 (1.3) | 181 (34.2) | 63 (47.4) | 32 (57.1) | <0.001 |
| Muscarinic antagonists^†^ | 23 (0.6) | 120 (22.7) | 11 (8.3) | 18 (32.1) | <0.001 |
| Glucocorticoids^†^ | 18 (0.4) | 58 (11.0) | 27 (20.3) | 13 (23.2) | <0.001 |
| Systemic adrenergic agonists | 1 (0.0) | 9 (1.7) | 0 (0.0) | 0 (0.0) | <0.001 |
| Other drugs | 4 (0.1) | 28 (5.3) | 0 (0.0) | 3 (5.4) | <0.001 |
| Diabetes medications^§^ |  |  |  |  |  |
| Biguanides | 862 (51.5) | 113 (50.7) | 28 (45.2) | 13 (56.5) | 0.744 |
| DPP-4 inhibitors | 270 (16.1) | 27 (12.1) | 11 (17.7) | 2 (8.7) | 0.327 |
| GLP-1 analogues | 16 (1.0) | 4 (1.8) | 0 (0.0) | 1 (4.4) | 0.225 |
| Sulfonylureas | 370 (22.1) | 51 (22.9) | 10 (16.1) | 7 (30.4) | 0.515 |
| Insulin | 454 (27.1) | 63 (28.3) | 17 (27.4) | 6 (26.1) | 0.986 |
| Data are presented as mean ± SD or median (IQR) for continuous measures and n (%) for categorical measures.  HR – heart rate; bpm – beats per minute; SBP – systolic blood pressure; BMI – body mass index; HF – heart failure; KCCQ TSS – Kansas city cardiomyopathy questionnaire; total symptom score; CSS – clinical summary score; OSS – overall summary score; NYHA – New York heart association classification; NT-proBNP – N terminal pro B-type natriuretic peptide; eGFR – estimated glomerular filtration rate; ACEI – angiotensin converting enzyme inhibitor; ARB – angiotensin receptor blocker; ARNI – angiotensin receptor blocker-neprilysin inhibitor; PCI – primary coronary intervention; CABG – coronary bypass surgery; CRT – cardiac resynchronization therapy; ICD – implantable cardioverter defibrillator.  *4 patients excluded  †Inhaled.  ^‡^ In combination with corticosteroids /antimuscarinics/ other drugs.  ^§^Only in patients with a medical history of diabetes (1983). | | | | | |
|  |  |  |  |  |  |
|  |  |  |  |  |  |

**Online Figure 1:**


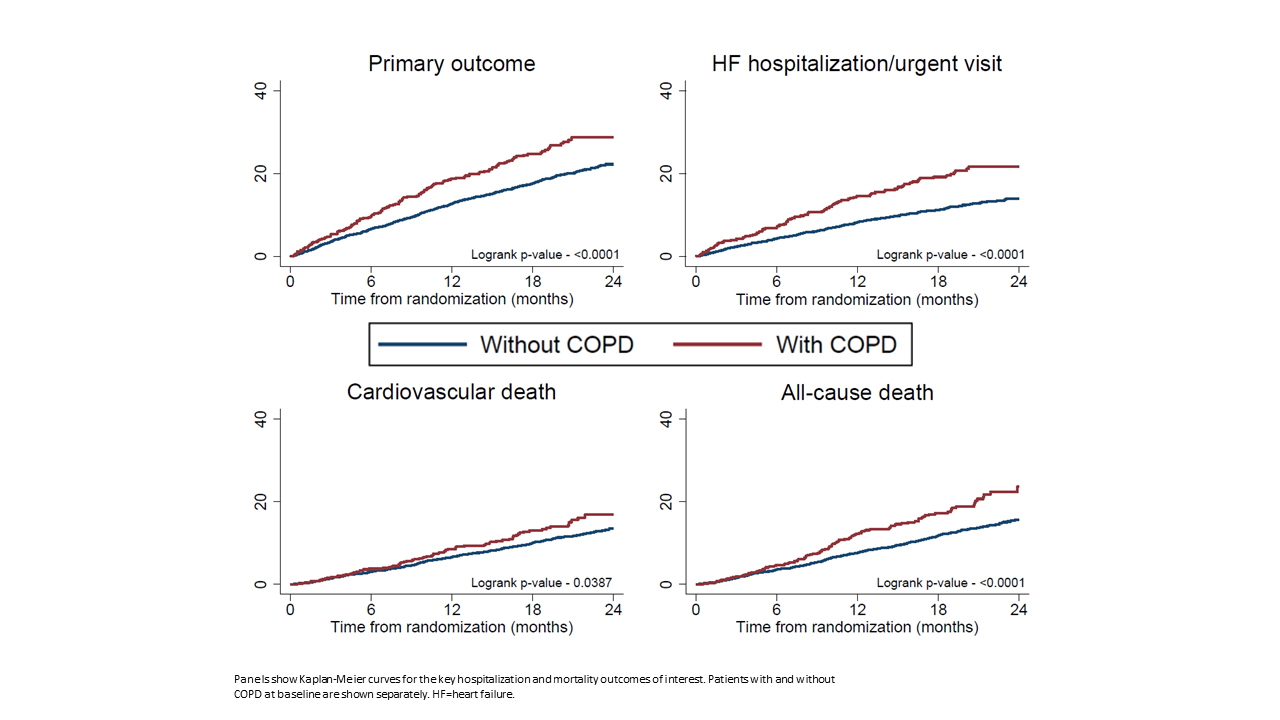


**Online Figure 2:**

Causes of death according to COPD status.


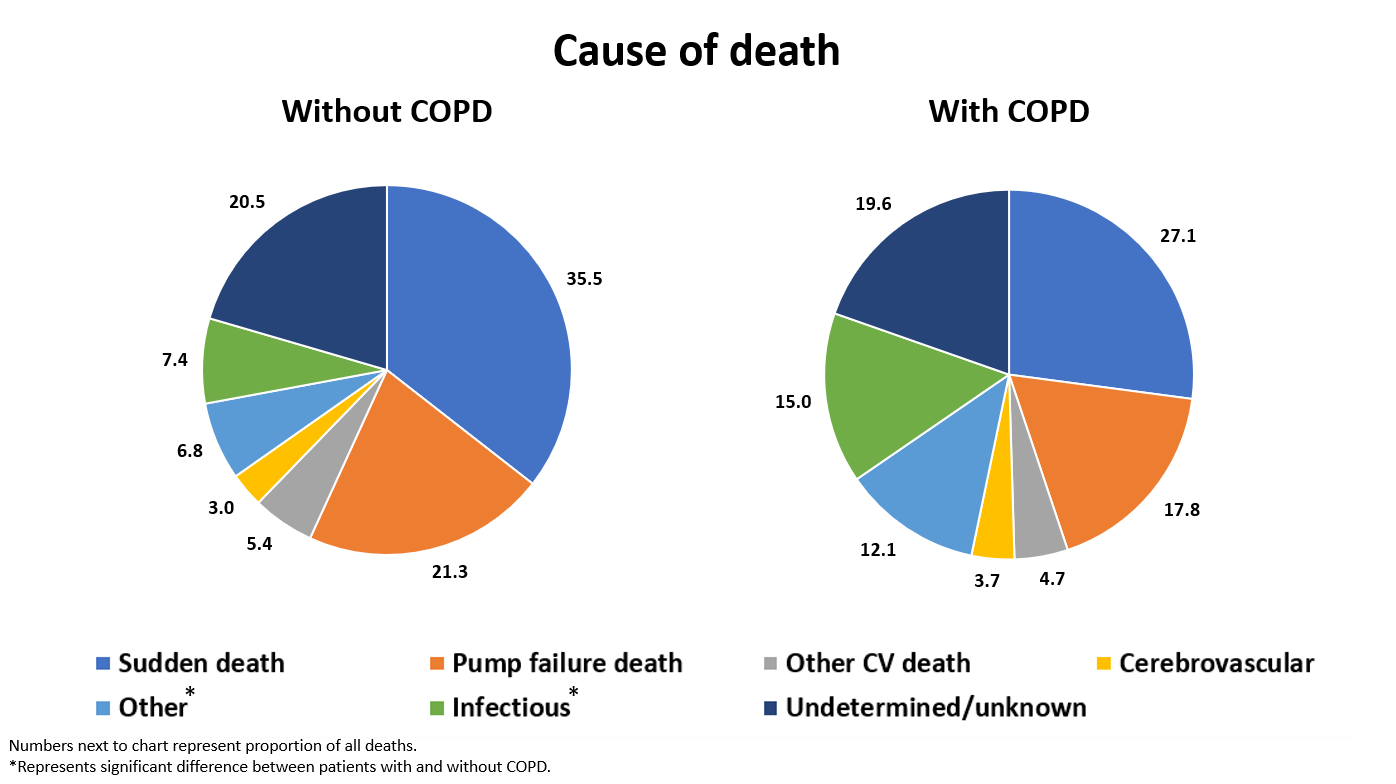


Other deaths: GI causes, Non-haemorrhagic/ stroke bleeding, hepatobiliary, malignancy, other, pancreatic, pulmonary failure, renal failure, suicide, trauma.

**Online Figure 3:** Clinical outcomes according to COPD and asthma status*


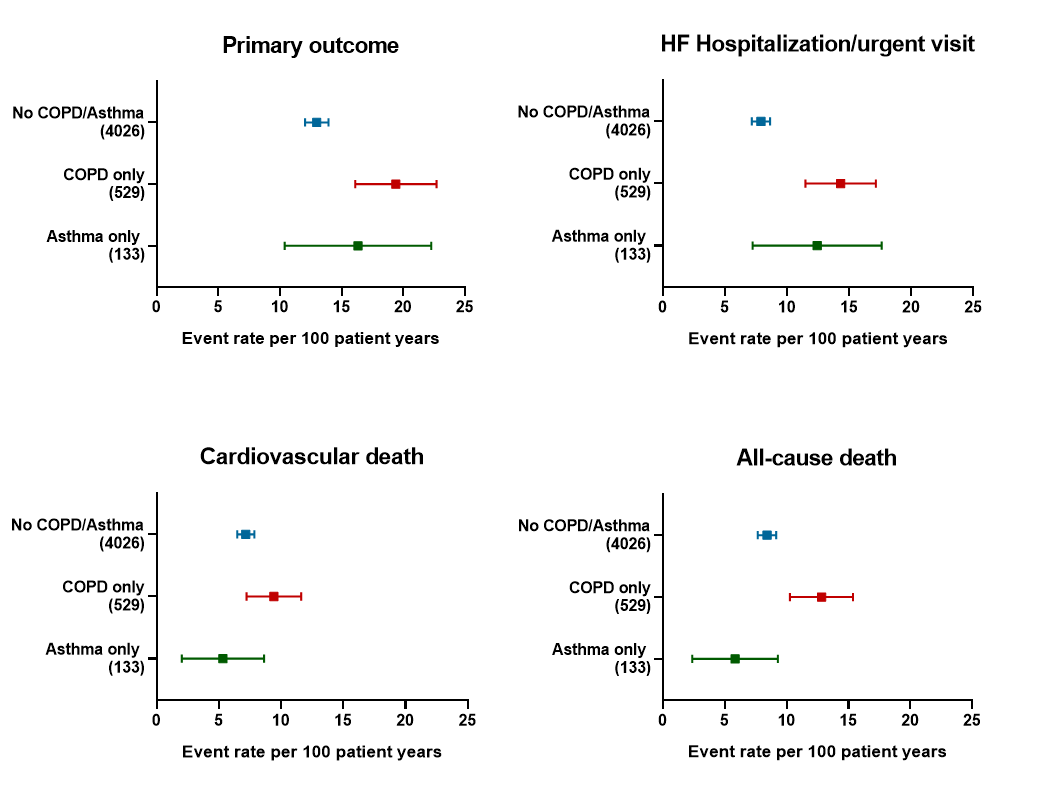


*This figure does not include 56 patients who had both investigator-reported COPD and asthma. Multivariable adjustment for variables including age, sex, smoking status and aetiology did not minimize the higher risk of all-cause death in patients with COPD only.
